# Inadvertent Inguinal Sarcoma Excision during Hernia Surgery: Outcomes, Gender Analysis, and Prevention

**DOI:** 10.1155/2020/8374790

**Published:** 2020-12-07

**Authors:** Joshua M. Lawrenz, James P. Norris, Marcus C. Tan, Eric T. Shinohara, John J. Block, Elizabeth J. Davis, Vicki L. Keedy, Jennifer L. Halpern, Ginger E. Holt, Herbert S. Schwartz

**Affiliations:** ^1^Department of Orthopaedic Surgery, Vanderbilt University Medical Center, Nashville, TN 37232, USA; ^2^Department of Orthopaedic Surgery, Spartanburg Regional Healthcare System, Spartanburg, SC 29303, USA; ^3^Department of Surgery, Vanderbilt University Medical Center, Nashville, TN 37232, USA; ^4^Department of Radiation Oncology, Vanderbilt University Medical Center, Nashville, TN 37232, USA; ^5^Department of Radiology, Vanderbilt University Medical Center, Nashville, TN 37232, USA; ^6^Department of Medicine, Vanderbilt University Medical Center, Nashville, TN 37232, USA

## Abstract

**Introduction:**

Inadvertent excision of a soft tissue sarcoma during hernia surgery is a preventable clinical scenario that leads to unnecessary patient morbidity. Prior series are few, which only include male patients with little focus on prevention. The purpose of this study is to report the presenting features and outcomes of both male and female patients who underwent inadvertent inguinal sarcoma excision during hernia surgery.

**Methods:**

A retrospective analysis of a single sarcoma referral center identified 33 patients who were referred for definitive treatment. Patients were divided into three clinically relevant groups based on intraoperative diagnosis, sex, and location of the mass relative to the inguinal ligament. *T*-tests and Fisher's exact tests were performed to compare continuous and categorical variables, respectively. Kaplan–Meier modeling was performed to assess sarcoma-specific survival.

**Results:**

Females were younger (47 years vs. 61 years, *p*=0.003) and had smaller sarcomas (6.7 cm vs. 11 cm, *p*=0.012) compared to males. Only two sarcomas (2/33, 6%) were <4 cm in size. The majority of sarcomas in females were above the inguinal ligament (12/14, 86%). Twenty-nine (88%) underwent definitive R0 excision. The mean number of surgeries per patient was three (range 1–13), with nineteen (58%) patients requiring flap reconstruction and six (18%) requiring vascular bypass. Five patients locally recurred (15%) at a mean of 38 months after definitive excision (range 5–128 months). Overall sarcoma-specific disease-free survival was 64%, with no difference between males (80 ± 11%) and females (59 ± 17%) (*p*=0.885). Mean follow-up was 75 months (range 5–212).

**Conclusion:**

This is the second largest study regarding inadvertent inguinal sarcoma excision and the first to include females. When a suspected hernia is >4 cm, irreducible, firm, and is growing, especially in females, consider obtaining preoperative advanced three-dimensional imaging (CT or MRI) that can differentiate a neoplasm from a hernia.

## 1. Introduction

In 1983, Joyce and Mankin introduced the term “*caveat arthroscopos*” or caveat arthroscopy describing ten patients referred to an orthopaedic oncology center after an untoward knee arthroscopy was performed for an unsuspected, extra-articular bone or soft tissue neoplasm [1]. In each case, the neoplasm was not appreciated preoperatively due to a variety of factors: lack of imaging, lack of recognition, inadequate exam, or inadequate history taken. Unfortunately, many patients had transsynovial biopsies of the lesions resulting in virgin compartment tumor contamination.

Inadvertent hernia surgery of a soft tissue sarcoma can be similarly termed caveat herniorrhaphy (CH). In this scenario, the patient is taken to the operating room for an inguinal hernia repair, and intraoperatively, an unexpected mass is encountered that is confirmed on final pathology to be a soft tissue sarcoma. Sarcomas comprise less than 1% of all adult malignancies [2], whereas inguinal herniorrhaphy is estimated to be the most common general surgical procedure in industrialized countries [3]. It is not uncommon for a mass to be incidentally discovered during herniorrhaphy, though the vast majority are benign entities, such as fat-containing hernias or cord lipomas [4, 5]. Given the routine nature of surgical exploration for a groin mass presumed to be a hernia, a soft tissue sarcoma masquerading as such is statistically at the bottom of the differential diagnosis. This naturally contributes to the potential for unplanned, incomplete excision of a soft tissue sarcoma in this setting. As a result, this unfortunate occurrence can induce significant morbidity on the patient, consisting of higher local recurrence rates, and the need for additional surgeries including plastic surgery reconstruction [6, 7].

Current literature is limited to a few case series evaluating the oncologic outcomes of inguinal sarcomas after reexcision. The three largest studies to date consist of 48, 21, and 14 patients from the same institution [5, 8, 9]. The importance of referral to a sarcoma center after CH and the role of multidisciplinary wide reexcision surgery as definitive management remain key conclusions. Little has been described however regarding the initial presentation of these patients, such as the initial intraoperative findings, surgical team, and location of the mass prior to referral. As well, there is a lack of formal description of this clinical scenario in females, as these studies almost exclusively consist of spermatic cord sarcomas.

We therefore sought to (1) describe the presenting demographic features of patients who had inadvertent, incomplete excisions of inguinal sarcomas during hernia surgery; (2) analyze clinically relevant features including intraoperative diagnosis, patient sex, and tumor location; and (3) report the surgical, oncologic, and survival data.

## 2. Patients and Methods

### 2.1. Patients

**Figure 1:**
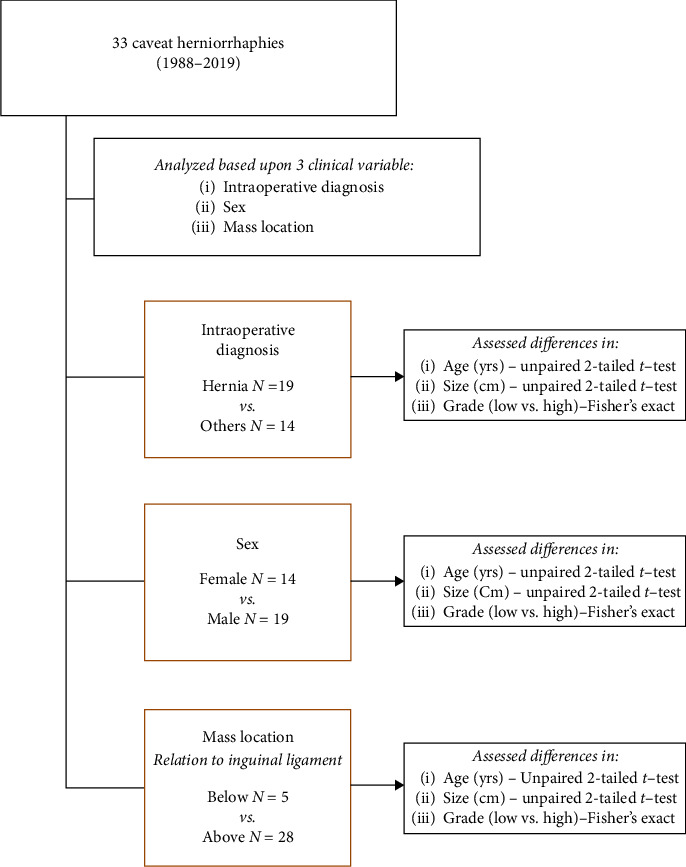
Study groups and design.

Following institutional review board approval, a retrospective analysis of a single sarcoma referral center between January 1988 and February 2019 was performed. Adult patients >18 years of age who were treated at our institution for a diagnosis of primary sarcoma located in the inguinal region, scrotum, perineum, or groin that was incompletely excised prior to presentation during a herniorrhaphy procedure were included. Patients were also excluded if they had metastatic disease to inguinal lymph nodes. There were 33 patients identified who met inclusion criteria. Patients were then divided into three clinically relevant groups based on the following: intraoperative diagnosis (hernia (*N* = 19) vs. others (*N* = 14)), sex (male (*N* = 19) vs. female (*N* = 14)), and location of the mass relative to the inguinal ligament (above (*N* = 28) vs. below (*N* = 5)). Patient groups can be found in Figure 1.

### 2.2. Data

Demographic, treatment, and clinical follow-up data were manually extracted from the electronic medical record. The following variables were collected: age, sex, tumor size, tumor grade, tumor histology, tumor location, intraoperative diagnosis (initial operative report), initial surgical team, perioperative radiation, number of surgeries (including flap reconstructions, vascular bypass procedures, and amputations), margin status after reexcision, local recurrence, distant metastasis, and overall and disease-free survival. We performed statistical analysis on the following variables: age, sex, tumor size, tumor grade, tumor location, and intraoperative diagnosis. Reexcision procedures were performed in a multidisciplinary fashion by fellowship-trained sarcoma specialty surgeons at our institution, including orthopaedic oncology, surgical oncology, urology, and gynecologic oncology. Reconstructive procedures were performed by vascular and plastic surgery.

### 2.3. Statistical Analysis

MedCalc Statistical Software (version 19.1.3) was used for all data analyses. Unpaired, two-tailed *t*-tests were performed to compare continuous variables (age and size). Fisher's exact test with two-tailed *p* values was performed to compare categorical variables (sex, diagnosis, location, and grade). *p* values less than 0.05 were considered significant. Kaplan–Meier modeling was performed to assess sarcoma-specific survival, and a log-rank test was used to compare male and female cohorts.

## 3. Results

### 3.1. Patient Demographics

There were 33 patients in our study cohort, including 19 males and 14 females. The mean age was 54 years (range, 21–81). Though all patients went to the operating room for a herniorrhaphy, a diagnosis of hernia was maintained intraoperatively in 19 patients (58%). The intraoperative diagnosis was changed in 14 patients (11 of which were female) to entities such as lymph node (7/33, 21%), scar (2/33, 6%), cyst (2/33, 6%), and hematoma (1/33, 3%). General surgery performed the majority of the initial surgical procedures (23/33, 70%), followed by urology (7/33, 21%), gynecology (2/33, 6%), and vascular surgery (1/33, 3%). Thirty-one patients (94%) had a mass size greater than 4 cm, and the mean mass size was 9.2 ± 5.1 cm. The majority of masses were located above the inguinal ligament (28/33, 85%) and were of high grade (26/33, 79%). Liposarcomas were found in 14 patients (42%), with the most common specific histology being a dedifferentiated liposarcoma in nine patients (27%). One patient was referred to our center with the newly developed metastatic disease 35 months after initial inadvertent excision. Of note, no patient was found to have had any preprocedure (prior to inadvertent excision surgery) three-dimensional advanced imaging (CT scan, MRI). Demographic data are shown in Table 1.

### 3.2. Analysis of Clinically Relevant Features

Significant differences were only found when comparing males and females. Females were younger (47 ± 13 years vs. 61 ± 12 years, *p*=0.003) and had smaller soft tissue sarcomas (6.7 ± 3.2 cm vs. 11 ± 5.5 cm, *p*=0.012) compared to males. As well, an intraoperative diagnosis of a hernia during initial surgery was far less frequent in females (3/14, 21% vs. 16/19, 84%, *p*=0.005). Tumor grade and mass location were no different between males and females. There were no differences in tumor size, patient age, and tumor grade based on intraoperative diagnosis or mass location. Statistical analyses are shown in Table 2.

### 3.3. Surgical, Oncologic, and Survival Data

The mean number of surgeries per patient was three (range 1–13). All patients initially had incompletely excised sarcomas with positive margins. Twenty-nine patients (88%) ultimately underwent definitive R0 excision: 24 patients upon first attempt, three patients upon second attempt, and two patients upon third attempt. Of the four remaining patients who did not undergo eventual R0 reexcision, one patient refused reexcision surgery and underwent chemoradiation, one underwent R1 reexcision surgery followed by the rapid development of metastases, and two underwent R1 reexcision followed by radiation therapy. Twenty-four (73%) patients underwent either neoadjuvant or adjuvant radiation therapy associated with definitive reexcision surgery. Nineteen (58%) patients required flap reconstruction, six (18%) required vascular bypass, and one (3%) eventually required amputation.

**Figure 2:**
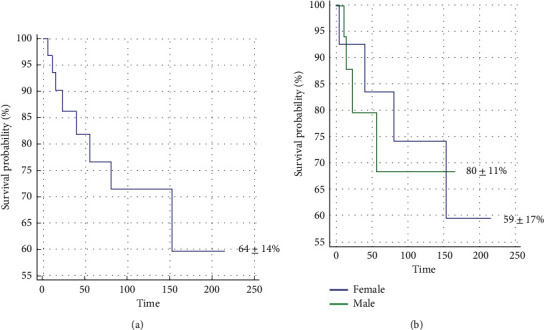
Kaplan–Meier survival curves. The graphs show Kaplan–Meier survival curves for the (a) entire cohort and for (b) males and females. (a) Overall sarcoma-specific survival in the entire cohort of 33 patients who underwent incomplete excision of an inguinal sarcoma was 64 ± 14% (time in months). (b) A comparison of sarcoma-specific survival curves between (1) females (59 ± 17%) and (2) males (80 ± 11%) who underwent incomplete excision of an inguinal sarcoma revealed no difference in long-term prognosis (*p*=0.885) (time in months).

Mean follow-up was 75 months (range 5–212). Five (15%) patients had a local recurrence at a mean of 38 months (range 5–128 months) after definitive excision. Seven (21%) patients developed new metastatic disease at a mean of 33 months after definitive excision (range 1–146), and eight (24%) died from their disease at a mean of 57 months after definitive excision (range 1–162). The overall sarcoma-specific survival rate was 64 ± 14% at the final follow-up, with no difference between males and females (80 ± 11% vs. 59 ± 17%, *p*=0.885). Surgical and oncologic data are shown in Table 3, and Kaplan–Meier survival curves are shown in Figure 2.

## 4. Discussion

Inadvertent sarcoma excision during a hernia surgery, termed caveat herniorrhaphy, results in unnecessary patient morbidity, consisting of major reexcision surgery and often reconstruction. A series from Memorial Sloan Kettering of spermatic cord sarcomas revealed 67% of their cohort was referred after prior incomplete excision, demonstrating the present and past dilemma in the initial recognition of sarcoma of the inguinal region [9]. The authors emphasized the correlation between negative margin reexcision surgery with reduced rates of local recurrence and improved disease-free survival. To our knowledge, the present study is the second largest series describing caveat herniorrhaphy and the first to report this entity in female patients. We sought to analyze pertinent clinical features at the initial presentation, with a particular emphasis on comparing male and female cohorts, in an effort to better understand how to prevent CH. Our data demonstrate that female patients were younger and had smaller soft tissue sarcomas than males and also were more likely to have a change in intraoperative diagnosis during initial excision surgery. Further, local recurrence rates and 10-year survival after wide reexcision surgery were similar to prior series, with no prognostic difference between males and females.

We primarily sought to describe and analyze clinically relevant presenting features of these patients at the time of CH. It was surprising to learn that 30% of initial herniorrhaphy procedures were carried out by surgical specialties other than general surgery, demonstrating the application of these findings across multiple disciplines. Furthermore, an in-depth review of operative reports revealed a difference in preoperative and postoperative diagnosis based on intraoperative findings in 42% of our cohort. Notably, near 80% of female patients left the operating room with a diagnosis other than hernia (such as lymph node or cyst), which inherently calls into question the basis for their preoperative diagnosis of a hernia. Unfortunately, clinical notes prior to initial surgery were sporadically available, preventing us from drawing further conclusions. In addition, all but two patients (31/33, 94%) had a tumor size >4 cm, with female patients having smaller tumors than men on average. It is important to consider that large deep masses may present clinically as small herniating masses (particularly liposarcomas, which were the most common histology in our cohort) which only places further emphasis on the importance of a thorough preoperative physical examination [10]. It remains unclear how to interpret the younger age of females compared to males in our cohort, as this is not a consistently described demographic difference seen in sarcoma. Lastly, there was no difference between males and females in regard to mass location relative to the inguinal ligament, as 84% and 86%, respectively, were above the inguinal ligament. It is interesting to note in females that despite the majority of their masses occurring above the inguinal ligament, they were still taken for a herniorrhaphy, when it is well described that femoral hernias (defined as occurring below the inguinal ligament) are far more likely in females compared to inguinal hernias (occurring above the inguinal ligament) [11].

**Figure 3:**
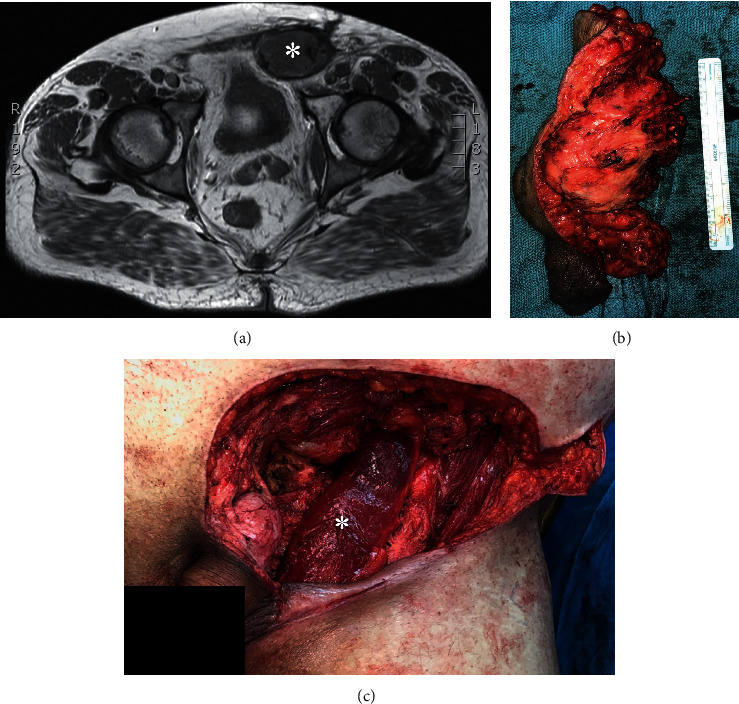
Case example: inguinal sarcoma reexcision and reconstruction. The images (a–c) and photos are of a 57-year-old male with a high-grade soft tissue sarcoma of the left groin who was referred to our institution after incomplete inguinal sarcoma excision during hernia surgery. (a) Axial T1 images of a pelvic MRI prior to reexcision surgery demonstrate a subfascial solid mass (*∗*) of the left inguinal region. (b) Wide reexcision specimen is shown, which included abdominal wall resection and left hemiscrotectomy/orchiectomy performed with multidisciplinary coordination between orthopaedic oncology, surgical oncology, and urology. (c) The resultant left groin soft tissue defect was covered with a rotational gracilis flap (*∗*) to protect the iliac vessels, and an acellular dermal matrix strattice with local tissue rearrangement (not shown).

The surgical, oncologic, and survival data in our cohort parallel prior series. The majority of patients required at least two additional surgeries after CH. Reexcision surgery for many consisted of major abdominal wall resection, including orchiectomy and hemiscrotectomy for males. As well, over half (58%) required flap reconstruction by plastic surgery (see Figure 3). The necessity for additional surgeries and increased likelihood of plastic reconstruction is consistent with previous reports when comparing unplanned vs. planned excisions of soft tissue sarcomas [6, 7]. Of the 32 patients that underwent reexcision, 75% (24/32) achieved negative margin resection on first attempt, with five more patients achieving R0 excision with subsequent attempts. Though our local recurrence rate of 15% was lower than previously reported rates of 42–55%, sarcoma-specific survival rate was similar to these studies at 64% [5, 8, 9]. Of note, we are the first series to report no difference in long-term prognosis between males and females.

Despite these patients having acceptable long-term outcomes, it is our firm belief that the unfortunate clinical scenario of caveat herniorrhaphy necessitates more widespread educational efforts to improve the early recognition of a groin lump as a sarcoma. Our data reiterates the well-known mantra in musculoskeletal oncology regarding mass size that “any lump bigger than a golf ball (4.27 cm) should have a diagnosis prior to excision” [12, 13], as 94% of our cohort had a mass size of >4 cm. Further, the importance of preoperative physical exam cannot be overstated. A benign groin hernia often presents as a painful soft lump or fullness in the groin region that can worsen with intra-abdominal strain (lifting, coughing, and Valsalva) and is often reducible [11], whereas suspicion for sarcoma ought to be raised with any painless, firm, irreducible mass that is >4 cm. Though we were unable to capture particular physical exam findings from the medical records prior to the initial surgery, we did confirm in all 33 cases that preoperative advanced three-dimensional imaging (CT or MRI) was not obtained. We have previously shown the significant correlation between incomplete excision in soft tissue sarcoma and lack of appropriate preoperative imaging [14].

**Figure 4:**
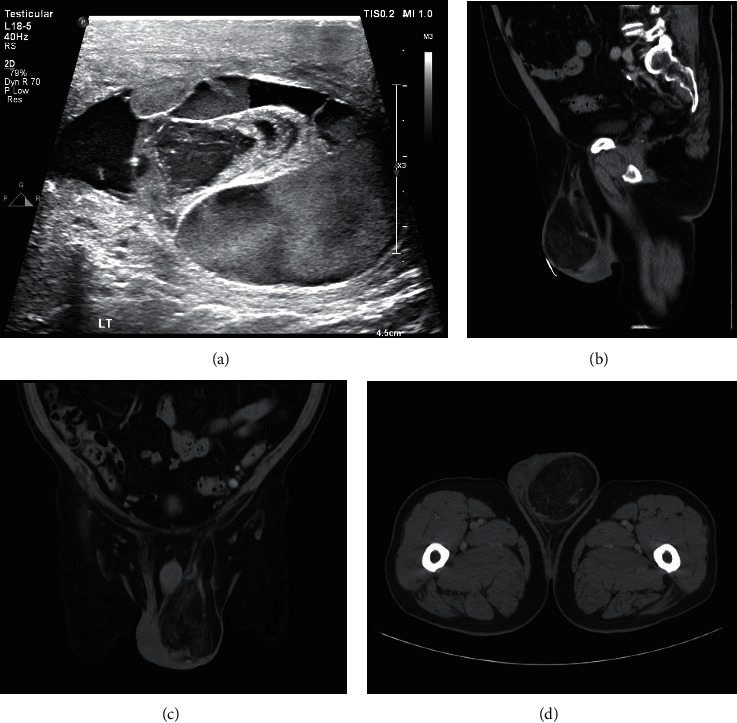
Case example: preoperative imaging comparison of ultrasound and CT scan. The images are of a 72-year-old male who presented with a firm testicular mass with associated scrotal swelling. (a) A scrotal ultrasound shows a mixed echotexture heterogeneous lesion within the left scrotum. Unfortunately, sonographic evaluation of inguinal or scrotal lesions lacks specificity in distinguishing fat- or bowel-containing inguinal hernia from a soft tissue sarcoma, such as a liposarcoma. (b–d) Sagittal, coronal, and axial contrast-enhanced preoperative CT images in the same patient demonstrate a predominately low-attenuation fatty 10 cm mass lesion within the inferior left inguinal canal and left scrotum. The presence of a thickened capsule around the periphery of the lesion as well as prominent enhancing internal septations with nodularity increases suspicion of a well-differentiated liposarcoma.

There are currently no specific guidelines available to recommend the best imaging modality of a groin mass at initial evaluation. In a review of 21 patients who underwent CH, Coleman et al. suggested ultrasound as the primary means to evaluate a solid groin mass [8]. A 2019 recent review of lesions presenting as abdominal and pelvic hernias also argued the efficacy of ultrasound to initially exclude clearly benign lesions such as cysts, bursas, or hernias [10]. They further argue all dimensions of the lesion must be entirely viewable; otherwise, more advanced imaging such as a CT scan or MRI is indicated. Not only do CT scan and MRI include a greater area of view, but they have been shown to be superior to ultrasound in detecting and characterizing fat, which may figure prominently as the most common inguinal sarcoma is a liposarcoma (see Figure 4) [15–17].

The added merit of MRI over CT scan has been suggested for its ability to detect small areas of macroscopic fat and suspicious areas of nodularity on postcontrast images that may signify dedifferentiation, in the case of liposarcoma [10, 17]. Thus, our recommendation in the case of a firm, irreducible, or growing mass >4 cm is advanced three-dimensional imaging be obtained (CT scan or MRI). If a CT scan is obtained and any findings are worrisome for a sarcoma, then an MRI with contrast is recommended. This should be especially considered in the case of a female with a presenting mass or lump above the inguinal ligament.

The limitations of this study stem from its retrospective nature and small dataset. The overwhelming majority of the patients in this cohort were referred from outside our health system, with many having relevant medical records unavailable for our review (i.e., preoperative ultrasound status and preoperative physical exam findings) that could further aid our understanding of this clinical scenario. Further, we were only able to analyze select variables given the small cohort size.

## 5. Conclusion

This is the second largest series describing caveat herniorrhaphy and the first to report this entity in female patients. Female patients were younger, had smaller soft tissue sarcomas than males, and also were more likely to have a change in intraoperative diagnosis during initial excision surgery. We found no difference in prognosis between males and females, with acceptable rates of local recurrence and long-term survival after wide reexcision surgery. Preoperative physical exam and advanced imaging remain paramount to differentiating a neoplasm from a hernia and in preventing the unfortunate dilemma and downstream morbidity of incomplete sarcoma excision.

## Figures and Tables

**Table 1:** Demographics data.

	Number (%)
Total cohort	33 (100)
Mean age, years (range)	54 (21–81)
Gender	
Male	19 (58)
Female	14 (42)
Intraoperative diagnosis	
Hernia	19 (58)
Others	14 (42)
Lymph node	7 (21)
Scar	2 (6)
Cyst	2 (6)
Unknown	2 (6)
Hematoma	1 (3)
Surgical specialty performing initial surgery	
General surgery	23 (70)
Urology	7 (21)
Gynecology	2 (6)
Vascular surgery	1 (3)
Tumor size	
<4 cm	2 (6)
>4 cm	31 (94)
Mean size, cm (SD)	9.2 (5.1)
Location	
Above inguinal ligament	28 (85)
Below inguinal ligament	5 (15)
Tumor grade	
High	26 (79)
Low	7 (21)
Tumor histology	
Dedifferentiated liposarcoma	9 (28)
Undifferentiated pleomorphic sarcoma	5 (15)
Well-differentiated liposarcoma	3 (9)
Synovial sarcoma	2 (6)
Solitary fibrous tumor	2 (6)
Malignant peripheral nerve sheath tumor	2 (6)
Liposarcoma	2 (6)
Myxofibrosarcoma	2 (6)
Epithelioid sarcoma	1 (3)
Angiosarcoma	1 (3)
Dermatofibrosarcoma protuberans	1 (3)
Peripheral neuroendocrine tumor	1 (3)
Rhabdomyosarcoma	1 (3)
Leiomyosarcoma	1 (3)

**Table 2:** Analysis of clinically relevant features.

	Intraoperative diagnosis		Sex		Mass location	
	Hernia (*N* = 19)	Others (*N* = 14)	Female (*N* = 14)	Male (*N* = 19)	Below inguinal ligament (*N* = 5)	Above inguinal ligament (*N* = 28)
Tumor size (cm)						
Mean (SD)	9.6 (5.3)	8.8 (5.0)	6.7 (3.2)	11 (5.5)	12 (7.5)	9 (4.6)
*p* value	0.678		*0.012*		0.193	
Patient age (years)						
Mean (SD)	57 (14)	52 (15)	47 (13)	61 (12)	48 (15)	56 (14)
*p* value	0.371		*0.003*		0.281	
Tumor grade (*N*)						
High grade	14	12	11	15	5	21
*p* value	0.670		1.000		0.559	

Additional analysis by sex			Sex		*p* value	
			Female (*N* = 14)	Male (*N* = 19)		
Intraoperative diagnosis (*N*)	Hernia		3	16	*0.005*	
	Others		11	3	*0.005*	

Mass location (*N*)	Above inguinal ligament		12	16	1.000	
	Below inguinal ligament		2	3	1.000	

**Table 3:** Surgical and oncologic data.

	Number (%)
Total cohort	33 (100)
Surgical	
Surgeries/patient, mean (range)	3 (1–13)
Flap reconstructions	19 (58)
Vascular bypass	6 (18)
Amputation	1 (3)
Oncologic	
R0 resection	29 (88)
Local recurrence	5 (15)
Metastasis	7 (21)
Mortality	8 (24)
Follow-up, months (range)	75 (5–212)

## Data Availability

The data are readily available at the author's institution for review in its original format.
